# Autonomic dysfunction in patients with advanced cancer; prevalence, clinical correlates and challenges in assessment

**DOI:** 10.1186/1472-684X-11-3

**Published:** 2012-03-01

**Authors:** Carol A Stone, Rose Anne Kenny, Brid Nolan, Peter G Lawlor

**Affiliations:** 1 Our Lady's Hospice and Care Services, Dublin 6w, Ireland; 2 Department of Gerontology, Trinity College Dublin, Dublin, Ireland; 3 Centre of Excellence for Successful Ageing, St James' Hospital, Dublin, Ireland; 4 Palliative Care Unit, Bruyere Continuing Care, Ottawa, Canada; 5 Division of Palliative Care, University of Ottawa, Ottawa, Canada

## Abstract

**Background:**

The results of a small number of studies of autonomic function in patients with advanced cancer suggest that autonomic dysfunction (AD) is common. In other disease-specific groups this is associated with decreased survival, falls and symptoms such as postural hypotension, nausea, early satiety and fatigue. The contribution of AD to symptoms in advanced cancer is unknown.

**Methods:**

We conducted a prospective cohort study designed to identify the risk factors for falls in patients with advanced cancer. Ambulant adult patients admitted consecutively to palliative care services were invited to participate. Participants underwent an assessment at baseline which included standard clinical tests of autonomic function, assessment of symptom severity, muscle strength, anthropometric measurements, walking speed, medication use, comorbidities and demographics. Information regarding survival was recorded ten months following cessation of recruitment. The clinical correlates of AD, defined as definite or severe dysfunction using Ewing's classification, were examined by univariate and multivariate logistic regression analysis. Survival analysis was conducted using Kaplan-Meier plots and the log rank test.

**Results:**

Of 185 patients recruited, 45% were unable to complete all of the clinical tests of autonomic function. Non-completion was associated with scoring high on clinical indicators of frailty. It was possible to accurately classify 138/185 (74.6%) of participants as having either definite or severe versus normal, early or atypical AD: 110 (80%) had definite/severe AD. In logistic regression analysis, age (OR = 1.07 [95% CI; 1.03-1.1] *P *= 0.001) and increased severity of fatigue (OR = 1.26 [95% CI; 1.05-1.5] *p *= 0.016) were associated with having definite/severe AD. In analysis adjusted for age, median survival of participants with definite/severe AD was shorter than in those with normal/early/atypical classification (χ^2 ^= 4.3, *p *= 0.038).

**Conclusions:**

Autonomic dysfunction is highly prevalent in patients with advanced cancer and is associated with increased severity of fatigue and reduced survival. Due to frailty, up to 45% of participants were unable to complete standard clinical tests of autonomic function. In order to further investigate the impact of AD and the therapeutic potential of treatment of AD in patients with advanced cancer, the validity of alternative novel methods of assessing autonomic function must be appraised.

## Background

The autonomic nervous system innervates blood vessels, the airways, intestines and urogenital organs and is largely under involuntary control. It regulates and coordinates bodily functions by effecting secretory activity of glands and contraction and relaxation of smooth and cardiac muscle [1]. Autonomic neuropathy may be idiopathic or occur as a complication of other conditions or as result of drugs. It is recognised as a common complication in patients with diabetes mellitus; for many it remains subclinical, with only a minority experiencing symptoms such as postural hypotension, nausea, vomiting and early satiety related to gastroparesis, nocturnal diarrhoea, bladder-emptying problems and male erectile dysfunction [2]. Autonomic dysfunction (AD) has also been shown to be a negative prognostic indicator following acute myocardial infarction and stroke [3]. The underlying mechanism is thought to be an increased risk of cardiac arrhythmias as a result of decreased vagal tone [4]. Although the autonomic nervous system innervates all organs, due to the serious implications of cardiovascular autonomic dysfunction, investigation of autonomic function involving the cardiovascular system has become most established [5].

Autonomic dysfunction has been described in patients with advanced cancer, in whom a high prevalence of AD is identified [6-9]. Postulated causes include decreased physical activity, treatment with vinca alkaloids or other medications, or paraneoplastic processes [10,11]. The precise contribution of AD to clinical findings and prognosis in advanced cancer is unclear. Only one study has demonstrated a relationship between AD and symptoms in patients with advanced cancer; Bruera *et al *found that patients with advanced breast cancer in whom all tests of cardiovascular autonomic function were abnormal were more likely to report symptoms of postural hypotension and chronic unexplained nausea [6,7,9]. More recent research on AD in advanced cancer has focussed on its prognostic significance; a small number of studies have identified a relationship between AD and shorter survival in advanced cancer [12-14].

Cardiovascular autonomic neuropathy has been shown to be a risk factor for falls in older adults with dementia [15]. We conducted a prospective study of the risk factors for falls in patients with advanced cancer. In view of the reported high prevalence of AD in patients with advanced cancer we elected to include tests of cardiovascular autonomic function in our research assessment. Autonomic function is most commonly measured by the application of a group of clinical tests, which aim to measure sympathetic and parasympathetic activity, by measuring end-organ responses to physiological perturbations [16]. Ewing *et al *devised a battery of four tests which generate three outcome measures of parasympathetic activity and two of sympathetic activity, the results of which can be used to grade the severity of autonomic dysfunction [17]. In this paper we specifically report our findings in relation to the frequency and clinical correlates of AD, highlight and evaluate the difficulties experienced in measuring autonomic function in patients with advanced cancer, and make recommendations regarding the direction of future research in this area.

## Methods

### Setting and participants

Eligible patients who were admitted consecutively to the palliative care services provided by Our Lady's Hospice and Care Services (November 24, 2008 - Dec 24, 2010) were invited to participate. The palliative care services consist of inpatient care provided in a 36-bed inpatient unit (IPU), a day hospice service and a home care service. Patients aged 18 years or older with a diagnosis of metastatic or loco-regionally advanced cancer were eligible for inclusion. Exclusion criteria were as follows: being unable to stand and mobilize unassisted, actively dying or considered too unwell by the admitting and research teams, registered blind, using continuous oxygen, and being aphasic or unable to converse in English. Eligible patients received written information on the study at the time of admission to services. Enrolment of patients with impaired cognition (Short Orientation-Memory Concentration Test (SOMCT) score greater than 11) required the assent of the patient in addition to consent from their proxy. The SOMCT error score ranges from 0-28; the normal score range is 0-6 [18]. All other participants provided informed consent. The study was approved by St. Vincent's University Health Group Ethics Committee.

### Data collection

Demographic details, comorbidities and medications were transcribed from admission notes and verified at patient interview. The following were considered to be cardioactive medications; beta blockers, tricyclic antidepressants, nitrates, rate-limiting calcium antagonists, anticholinergics and other antiarrhythmics. Performance status was recorded using the Palliative Performance Scale (PPS) [19].

All assessments were conducted between 0900 h and 1300 h. All participants were asked to refrain from smoking and caffeine ingestion on the morning of assessment, but were not asked to stop any of their usual medications or fast. A physician and research nurse performed the tests of autonomic function in a quiet room at ambient temperature (21-23 C). Autonomic function tests were carried out using a modified Ewing's battery [17]. Heart rate was measured by ECG using standard limb leads; heart rate (HR) tests were excluded if invalidated by arrhythmia, excessive ectopic activity or movement artefact. Blood pressure (BP) was monitored using the Finometer Pro device (Finapres Medical Systems BV, Amsterdam, the Netherlands) which enables noninvasive beat-to-beat BP measurement from finger arterial BP. The BP recordings are derived from the circumferential pressure generated by a finger cuff, which is varied to maintain a constant digital arterial size, as measured by a photoplethysmograph. Under such conditions the external cuff pressure equals the internal digital arterial pressure [20]. Participants rested in the supine position for at least ten minutes before testing. During this time they were covered with a blanket and wore a thermal mitten with glove liner in order to improve BP signal pick-up. Blood pressure tests were excluded if the trace was obscured by movement artefact or artefact due to external pressure on the finger-cuff.

#### Parasympathetic tests

1. Deep breathing

Whilst supine, participants were requested to 'take slow deep breaths, so that each breath in lasts five seconds and each breath out lasts 5 s, for a total of six consecutive breaths'. This was rehearsed prior to testing and the tester guided the timing of the breaths for the participant by verbally counting through each of the six breaths/cycles. The maximum and minimum HR during each breathing cycle was calculated from the corresponding shortest and longest R-R interval, and the response recorded as the mean of the differences during three successive breathing cycles.

2. Active stand

Participants were requested to stand up from the supine position as quickly as possible and to remain standing, in silence, for three minutes, with the monitored arm resting by their side. Assistance with rising was provided when this could not be achieved independently. Heart rate response was measured as the ratio of the maximum R-R interval at or around the 30^th ^beat after starting to stand, to the minimum R-R interval at or around the 15^th ^beat.

3. Valsalva manoeuvre

The valsalva manoeuvre was achieved by forced expiration, against an open glottis. Participants were requested to achieve a constant pressure of 40 mmHg for 15 s. The procedure was rehearsed prior to testing and the tester guided the participant by counting aloud through the fifteen seconds. The test was performed three times and the best response used for analysis. A minimum of one test achieving 30 mmHg for 12 s was required for inclusion. Heart rate response was taken as the ratio of the maximum R-R interval shortly after the manoeuvre to the minimum R-R interval during the procedure.

#### Sympathetic test

1. Active stand

The change in BP was measured as the difference between the baseline BP whilst supine and the lowest BP after standing.

Results for the valsalva manoeuvre were graded as normal or abnormal, and all other tests as normal, borderline or abnormal using the values recommended by Ewing *et al *[17]. Overall autonomic function was described using Ewing's classification system:

• Normal: all tests normal or one borderline

• Early dysfunction: one of the three HR tests abnormal or two borderline

• Definite dysfunction: two or more HR tests abnormal

• Severe dysfunction: two or more HR tests abnormal plus one BP test abnormal or both borderline

• Atypical pattern: any other combination of abnormal tests [17].

Severity of tiredness, nausea, loss of appetite and shortness of breath were measured using the Edmonton Symptom Assessment Scale; an 11 point numerical rating scale from 0-10, whereby larger numbers represent increased symptom severity [21]. Information regarding survival of study participants was obtained from an electronic palliative care patient administration database system, used by Our Lady's Hospice & Care Services and the hospitals within its catchment area. Survival in days was measured from the day of assessment. The analysis of survival times was conducted on Sept 24, 2011.

BMI was calculated from participants' height and weight as measured on the day of assessment, and weight loss by subtracting current weight from reported weight prior to cancer diagnosis. Walking speed was measured using the timed 'Up and Go' (TUAG) whereby the participant is asked to rise from a seated position, walk to a marked spot three metres away, turn around and return to their seat. The participant is instructed to walk at their normal pace and may use any gait aid normally used. Timing is started when the participant is instructed to 'go' and stopped when they are seated in the chair again [22]. Grip strength was measured 3 times in each hand using a hydraulic hand dynamometer (Jamar, Samons Preston Rolyan, Bolingbrook, IL). The result used was the best result of the six measurements.

### Statistical methods

Demographic details and clinical variables were summarised using descriptive statistics. Comparisons of groups of categorical variables were conducted using the Chi-squared test, of normally distributed continuous variables using the 2-sample t-test and of non-parametric variables using the Mann-Whitney U test. Variables shown to be associated with AD in univariate analyses, with *p *< 0.1, were entered into logistic regression models, using forwards and backwards stepwise variable entry. Only variables that were significant at *p *< 0.05 were retained in the model. The relationship between AD and survival was examined using survival analysis methods, including the log rank test.

## Results

During the study period, there were 1,607 admission episodes involving 1,117 individuals, of whom 693 (62%) were ineligible, 239 (21.4%) declined and 185 (16.6%) were recruited. There were no significant demographic differences between participants and those who declined (see Table 1).

**Table 1:** Demographic details of participants and those who declined participation

	Participants (n = 185)	Declined (n = 239)	Analysis
**Gender Male (%)**	52.4	50.2	χ^2 ^= 0.2, 1df p = 0.65

**Age (years)**	68 ± 12.6	68.3 ± 13.1	t = 0.12, *p *= 0.9

**Cancer diagnosis (%)**			

**Bronchial**	18.4	23.4	χ^2 ^= 5.7,
**Breast**	14.1	12.1	9df
**Lower GI**	14.1	13.8	*p *= 0.8
**Upper GI**	11.4	10.9	
**Prostate**	9.7	7.1	
**Gynaecological**	7	6.3	
**Pancreatic/hepatobiliary**	6.5	8.8	
**Urological not prostate**	5.4	4.6	
**Brain**	2.7	0.8	
**Other**	10.8	12.1	

### Completion of individual components of Ewing's battery

#### HR response to deep breathing

The HR data of 14/185 (7.6%) participants were invalidated by arrhythmia. A further nine participants were unable to complete three consecutive breaths according to the study protocol, due to inattention and/or difficulty understanding and retaining information. The median SOMCT error score in those who completed the test was 2 compared with 6.5 (*p *= 0.015) in those who did not complete it.

#### HR response to active stand

The HR data of 14/185 (7.6%) participants were invalidated by arrhythmia and one other by excessive artefact at the time of standing.

#### BP response to active stand

The BP data of 42/185 (22.7%) participants was invalidated due to failure to obtain a good quality trace or due to artefact; most commonly due to external pressure on the finger cuff at the time of the stand.

#### HR response to valsalva manoeuvre

Eighty-three (45%) participants were unable to complete the valsalva manoeuvre. We conducted analyses to explore our *post-hoc *hypothesis that the high prevalence of non-completion of the valsalva manoeuvre was due to the phenotypic characteristics of our study population. We observed that patients who had features consistent with the geriatric syndrome of *frailty *[23] were less likely to be able to complete the valsalva manoeuvre. See Table 2 for results. In view of the high prevalence of dyspnoea in advanced cancer we included the ESAS item on severity of shortness of breath in our analysis, but found that this was not associated with ability to complete the valsalva manoeuvre.

**Table 2:** Features of participants according to whether they were able to complete the valsalva manoeuvre

Variable	Mean/median Valsalva completed	Mean/median Valsalva not completed	*P *value
**Weight loss (Kg)**	4.3	7.8	0.09 (t = -1.7)

**BMI**	26	22.6	< 0.0001 (t = 4.9)

**TUAG (secs)**	14.06*	18.7*	< 0.0001

**Grip strength (kg)**	24.5*	18.0*	< 0.0001

**Tiredness (ESAS)**	3*	4*	0.2

**Age (years)**	65.4	71.5	0.001 (t = -3.3)

*Median value

### Prevalence of autonomic dysfunction and associated factors

**Figure 1:**
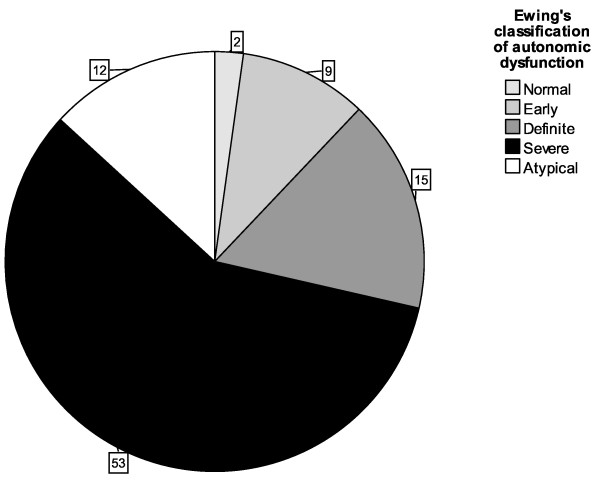
**Pie chart to show prevalence of autonomic dysfunction in patients with advanced cancer (n = 91)**.

Due to the high levels of missing data pertaining to the HR response to valsalva manoeuvre and BP response to active stand, it was only possible to accurately define autonomic function, using Ewing's classification (normal, definite, severe or atypical), for 91/185 (49.2%) participants. See Figure 1. By collapsing the Ewing's classification into a binary classification of definite/severe versus other, it was possible to accurately classify 138/185 (74.6%) participants as having either normal, early or atypical, (other category) versus definite or severe AD. Of 138 patients 110 (80%) had definite or severe AD. Having definite/severe AD was associated with poor performance status (χ^2 ^for trend = 8.2, *p *= 0.004) and increasing age; see Table 3 for contingency table of binary AFT classification according to age groups defined by quartiles (χ^2 ^for trend = 7.6, *p *= 0.006). In univariate analysis, gender, taking cardioactive medication and having a diagnosis of diabetes mellitus were not associated with binary AFT classification (χ^2 ^= 0.17, *p *= 0.7, χ^2 ^= 0.89, *p *= 0.4 and χ^2 ^= 0.4, *p *= 0.5 respectively), whereas having at least one cardiovascular comorbidity was associated with having definite/severe AD (χ^2 ^= 3.79, *p *= 0.05). Having definite/severe AD was associated with severity of tiredness as measured using the ESAS (median 4/10 versus 2/10, *p *= 0.006), but not with severity of appetite loss (median 3/10 versus 1/10, *p *= 0.07) or nausea (median 0/10 versus 0/10, *p *= 0.9). Age, PPS, taking cardioactive medications, severity of tiredness (ESAS) and severity of appetite loss (ESAS) were entered into the logistic regression models. However, only age (OR = 1.07 [95% CI; 1.03-1.1] *P *= 0.001) and severity of tiredness (OR = 1.26 [95% CI; 1.05-1.5] *p *= 0.016) were shown to be significantly associated with a diagnosis of definite or severe autonomic dysfunction.

**Table 3:** Binary AFT classification according to age (quartiles)

	Age Group					Total
	
		< 58 yrs	58-67 yrs	68-76 yrs	≥ 77 yrs	
**Normal/early/atypical AFT classification**	Count Expected count	14 6.3	5 7.3	3 7.1	6 7.3	28 28

**Definite/severe AFT classification**	Count Expected count	17 24.7	31 28.7	32 27.9	30 28.7	110 110

**Total**	Count Expected count	31 31	36 36	35 35	36 36	138 138

**Figure 2:**
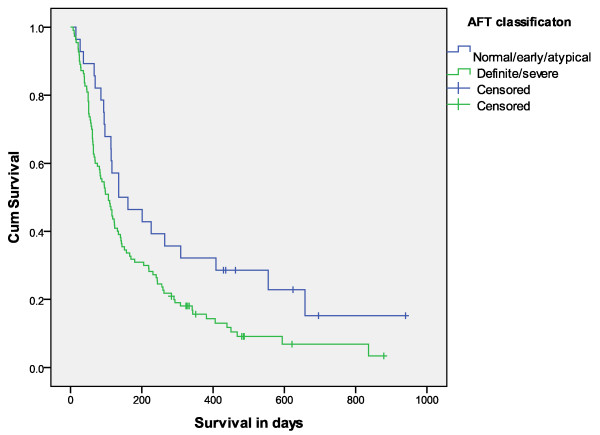
**Kaplan-Meier plot to show relationship between survival and autonomic function (n = 138)**.

The median survival for participants with definite/severe AD was 106 days (95% CI; 78.6-133.4) compared with 135 days (95% CI; 24.8-245.2) in those with normal/early/atypical classification (χ^2 ^= 4.8, *p *= 0.028). See Figure 2. The relationship between AD and survival persisted in analysis adjusted for age, defined by quartiles as above (χ^2 ^= 4.3, *p *= 0.038).

Eighty-four of the 143 participants (58.7%) who had valid active stand BP data had a systolic BP drop of at least 30 mmHg on standing.

## Discussion

Using Ewing's classification it was possible to diagnose the presence or absence of definite or severe AD in 138/185 (74.6%) participants, of whom 80% had definite/severe AD. This finding is consistent with the prevalence of moderate/severe AD measured in patients with advanced cancer (n = 50), as reported by Walsh and Nelson, and in men with advanced cancer (n = 48), as reported by Strasser *et al *of 82% and 81%, respectively [7,9]. In our study, severity of fatigue was greater in patients with definite/severe AD, and although ESAS scores for loss of appetite were greater in those with definite/severe AD, this did not reach statistical significance. Median ESAS scores for nausea were zero in both groups, which most likely reflects the availability of effective treatment for this symptom. Having definite/severe AD was associated with shorter survival. Although this confirms the findings of previous studies of AD in patients with advanced cancer, it has yet to be clarified whether AD causes shorter survival or if factors associated with shorter survival, such as increased time spent in bed, contribute to severity of AD.

A striking finding of our study was the poor feasibility of conducting autonomic function testing, using Ewing's battery, in patients with advanced cancer. Forty-five percent of patients were unable to complete the valsalva manoeuvre, despite our having adopted a lower threshold for the duration, pressure and number of tests completed, than is standard. The results of the *post hoc *analyses supported our observation that more frail patients were less likely to be able to complete the valsalva manoeuvre. Prior to the active stand, participants were requested not to grip anything with their right hand during the process of rising or during the three minute stand: most participants did receive the assistance of one of the testers to rise from the supine to seated position. Despite this many participants experienced difficulty with getting up quickly. Additionally, we took steps to ensure a good digital BP recording by keeping the participant warm prior to testing. Despite this, BP data from 23% of participants were invalid, mainly due to artefact from external pressure on the finger cuff at the time of standing or due to a poor quality trace.

Walsh and Nelson reported that participants in their study also had difficulty with rising from a supine position to standing, and that they found the valsalva manoeuvre stressful, but despite this 48/50 (96%) patients managed to complete it [7]. Bruera *et al *reported that 8/43 (18.6%) participants had missing HR and BP data for the active stand as they were unable to stand up readily. It is likely that our use of beat-to-beat BP measurement from finger arterial BP, though now the standard in clinical and research autonomic function laboratories, resulted in our relatively high rate of failure in obtaining active stand BP data in this patient population. All other studies in patients with advanced cancer measured BP at the brachial artery with a sphygmomanometer, which has the drawback of not providing continuous monitoring, but is less susceptible to artefact resulting from external pressure and peripheral vasoconstriction.

Our use of a modified version of the Ewing's battery of tests was a notable study limitation: we omitted a second test of sympathetic function, the BP response to isometric exercise, whereby the patient is instructed to grasp a dynamometer and sustain a fixed, isometric contraction for 3 min at 30% maximum effort. We omitted this test for pragmatic reasons: an accurate diagnosis of definite AD according to Ewing's classification can be made based on 3 HR tests; this test had the lowest rate of completion in Walsh and Nelson's earlier study, as participants found it difficult. Furthermore, this test has been shown to have low sensitivity and specificity, due to problems standardising muscular effort and variability in muscle afferent activity when tested in trained versus untrained muscles [7,16].

The ranges for normal values for each test were derived by Ewing from tests in healthy subjects aged 16-69 years [17]. However, HR responses to deep breathing, active stand and the valsalva manoeuvre decline with age. Our use of Ewing's normal values, rather than determining age-specific normal values from testing age-matched controls, may have resulted in false negative test results in younger patients and also means that we cannot isolate the effect of a diagnosis of advanced cancer from the effect of normal aging on AD. Despite this, 54.8% of those aged less than 58 years were classified as having definite/severe AD. Unlike previous studies, we did not exclude patients who had other medical conditions known to be associated with AD or those taking medications which may affect autonomic reflexes. However, we found that in the context of advanced cancer, these conditions do not significantly increase the risk of AD.

Autonomic dysfunction is common in patients with advanced cancer. The findings of Bruera *et al *suggest that AD may be associated with symptoms of postural hypotension and unexplained nausea [6]. In view of the high prevalence of orthostatic hypotension (OH) in this study, we recommend routine enquiry for symptoms suggestive of OH and measurement of lying and standing BP in patients with advanced cancer. Management of OH includes discontinuation of antihypertensive medication, adoption of physical counter- manoeuvres, and use of mineralocorticoid (e.g. fludrocortisone) and/or adrenergic agonist (e.g. midodrine) medications [24]. For patients with unexplained symptoms suspected to be due to AD involving other systems, targeted investigation, such as gastric emptying studies in patients with chronic unexplained nausea, should be considered [25].

In this study, patients with definite or severe AD had higher scores for severity of tiredness, as measured by the ESAS. Fatigue has been shown to be associated with AD in patients with Multiple Sclerosis and Primary Biliary Cirrhosis [26,27]. Impaired autonomic function has also been described in patients with Chronic Fatigue Syndrome (CFS) [28,29] and patients with vasovagal syncope (VVS) have been shown to have higher levels of fatigue than age-matched controls [30]. A postulated explanation for these associations is that fatigue occurs as a result of impaired organ perfusion related to hypotension [30]. Alternative explanations, in the case of CFS, are that AD develops as a result of reduced physical activity, or that both the fatigue and AD have a common aetiology. However, early studies in patients with CFS and VVS suggest that severity of fatigue may improve with treatment of AD related postural hypotension. In a cross-sectional study of patients with VVS diagnosed and treated by a single Falls and Syncope Service (n = 91), patients whose syncopal symptoms had responded to conventional treatment (conservative advice, followed three months later in the event of persistent symptoms, by commencement of either fludrocortisone, midodrine or a selective serotonin reuptake inhibitor) reported less severe fatigue and daytime sleepiness than those with persistent syncopal symptoms [30]. In a pilot study of 10 patients with CFS shown to have abnormal cardiovascular responses to a head-up tilt test, for 6 patients, treatment with midodrine resulted in normalisation of cardiovascular responses at three months, followed by an improvement in fatigue scores 4-8 weeks later [31]. Exploration of the relationship between autonomic dysfunction and fatigue, the potential for reversibility of AD with pharmacological intervention, and the impact of this on fatigue and survival in patients with advanced cancer, are all worthy of further investigation.

However, in view of our findings relating to the feasibility of conducting standard clinical tests of autonomic function in a large cohort of patients with advanced cancer, we recommend that the reliability and validity of novel methods of assessment of autonomic function are investigated further. The findings of Fadul *et al *suggest that measurement of heart rate variability (HRV) may provide a useful measure of AD in this population, for both research and clinical purposes. Power spectral analysis of heart rate fluctuations, recorded by continuous electrocardiogram, provide a simple and non-invasive technique for analysing autonomic function. In their study of men with advanced cancer, Fadul *et al *found a strong association between the results of the Ewing's battery and the time domain measure 'standard deviation of normal to normal beat interval' (r = 0.44*, p *= 0.002) [13].

## Conclusions

Autonomic dysfunction is highly prevalent in advanced cancer and is associated with severity of fatigue and shorter survival. Research findings in patients with VVS and CFS suggest that correction of autonomic dysfunction may result in alleviation of fatigue. Future research on the impact of AD and its treatment in patients with advanced cancer must also address the potential for novel methods of assessment of autonomic function to provide a reliable proxy for the standardised clinical tests; due to frailty, 45% of participants in this study were unable to complete Ewing's battery of tests emphasising the need to explore alternative methods for evaluation of autonomic function in this population.

## Competing interests

The authors declare that they have no competing interests.

## Authors' contributions

CS coordinated and designed the study, collected the data, performed the statistical analysis and drafted the manuscript. RAK conceived of the study, participated in its design and revised the manuscript critically for important intellectual content. BN helped to conduct the data collection and processing and to draft the manuscript. PGL conceived of the study, participated in its design and helped draft the manuscript. All authors read and approved the final manuscript.

## Pre-publication history

The pre-publication history for this paper can be accessed here:

http://www.biomedcentral.com/1472-684X/11/3/prepub
